# The CCR4–NOT complex suppresses untimely translational activation of maternal mRNAs

**DOI:** 10.1242/dev.201773

**Published:** 2023-10-18

**Authors:** Shou Soeda, Masaaki Oyama, Hiroko Kozuka-Hata, Tadashi Yamamoto

**Affiliations:** ^1^ Cell Signal Unit, Okinawa Institute of Science and Technology, Kunigami, 904-0495, Japan; ^2^ Laboratory for Cell Systems, Institute for Protein Research, Osaka University, Suita, 565-0871, Japan; ^3^ Medical Proteomics Laboratory, The Institute of Medical Science, The University of Tokyo, Minato-ku, 108-8639, Japan

**Keywords:** MRNA deadenylation, CCR4–NOT complex, Mouse oocyte, Translation

## Abstract

Control of mRNA poly(A) tails is essential for regulation of mRNA metabolism, specifically translation efficiency and mRNA stability. Gene expression in maturing oocytes relies largely on post-transcriptional regulation, as genes are transcriptionally silent during oocyte maturation. The CCR4–NOT complex is a major mammalian deadenylase, which regulates poly(A) tails of maternal mRNAs; however, the function of the CCR4–NOT complex in translational regulation has not been well understood. Here, we show that this complex suppresses translational activity of maternal mRNAs during oocyte maturation. Oocytes lacking all CCR4–NOT deadenylase activity owing to genetic deletion of its catalytic subunits, Cnot7 and Cnot8, showed a large-scale gene expression change caused by increased translational activity during oocyte maturation. Developmental arrest during meiosis I in these oocytes resulted in sterility of oocyte-specific *Cnot7* and *Cnot8* knockout female mice. We further showed that recruitment of CCR4–NOT to maternal mRNAs is mediated by the 3′UTR element CPE, which suppresses translational activation of maternal mRNAs. We propose that suppression of untimely translational activation of maternal mRNAs via deadenylation by CCR4–NOT is essential for proper oocyte maturation.

## Teaser

**Summary:** The CCR4–NOT complex is essential for proper oocyte maturation by suppressing translational activation of maternal mRNAs. Recruitment of CCR4–NOT to maternal mRNAs is mediated by the 3′UTR element CPE.

## INTRODUCTION

Post-transcriptional regulation is an essential mechanism for controlling gene expression in mammalian oocytes and early embryos (Passmore and Coller, 2022; Ozturk, 2019). Post-transcriptional regulation of mRNAs drastically changes upon meiotic resumption, which induces oocytes arrested at meiotic prophase to progress into meiosis-I prometaphase. Before resumption of meiosis, oocytes undergo a growth phase, and during this phase, they synthesize and store mRNAs, some of which are immediately translated for the growth process, whereas others remain dormant until the correct phase of development. Accumulated mRNAs form a supply of maternal mRNA that supports oocyte maturation in meiosis and several rounds of the cell cycle after fertilization. In mammals, fully grown oocytes are transcriptionally quiescent from before oocyte maturation until transcriptional activation, which occurs at the two-cell stage in mice and the four-cell stage in humans (Clift and Schuh, 2013). When oocytes enter the maturation phase, maternal mRNAs change drastically. Before oocyte maturation, maternal mRNAs are extremely stable; however, during oocyte maturation, maternal mRNAs undergo massive, yet selective, degradation. Meiotic resumption also triggers sequential translational activation of dormant maternal mRNAs. Therefore, dynamic regulation of both stability and translational activity of maternal mRNAs reflects the gene expression pattern during oocyte maturation.

Stability and translational activity of mRNAs are closely related to the lengths of their poly(A) tails. Shortening of mRNA poly(A) tails by deadenylation is the initial step of mRNA degradation. In contrast, long poly(A) tails are associated with mRNA stability and persistence (Garneau et al., 2007). Poly(A) tails also function as binding sites for cytoplasmic poly(A)-binding protein (Pabpc) (Smith et al., 2014; Baer and Kornberg, 1983), which stimulates translation by potentiating interaction of the translation initiation factor, eIF4G, with the cap-binding protein, eIF4E (Tarun and Sachs, 1996; Jacobson and Peltz, 1996). Cytoplasmic polyadenylation is one of the mechanisms for temporal control of gene expression at the post-transcriptional level. Changes in the interaction of RNA-binding proteins with target mRNAs mediated by sequence elements in their 3′ untranslated regions (UTRs), or with other proteins, can promote cytoplasmic polyadenylation, which enables expression of particular transcripts at the correct time. One well-characterized example is the regulatory mechanism mediated by cytoplasmic polyadenylation element (CPE) in mRNA 3′UTRs and CPE-binding protein (CPEB). When oocytes resume meiosis, CPEB is phosphorylated by several mitotic kinases, which leads to activation of cytoplasmic poly(A) polymerases interacting with CPEB (Oh et al., 2000; Vasudevan et al., 2006). CPE-containing maternal mRNAs thereby undergo cytoplasmic polyadenylation, leading to their translational activation (Richter, 2007).

The importance of translational control mediated by cytoplasmic regulation of poly(A) tail length has been reported in many types of cells, such as neuronal cells (Wu et al., 1998), cancer cells (Perez-Guijarro et al., 2016) and early embryonic cells. During oocyte maturation, cytoplasmic polyadenylation is essential for proper cell cycle progression. Polyadenylation/deadenylation at Metaphase-II (MII) arrest is one example. Mammalian oocytes arrest the cell cycle at MII after a hormone stimulus to prevent development without a paternal genome. The Moloney sarcoma oncogene (Mos)-extracellular signal-regulated kinase (Erk) pathway maintains activity of cyclin B and cyclin-dependent kinase 1 (Cdk1) (Haccard et al., 1993; Colledge et al., 1994; Hashimoto et al., 1994). Arrest at MII is achieved by suppression of anaphase-promoting complex (APC), thereby protecting cyclin B from destruction by APC. Factors responsible for this suppression are called cytostatic factors (CSFs) and the factor farthest downstream is endogenous meiotic inhibitor 2 (Emi2; Fbxo43) (Tung et al., 2005; Shoji et al., 2006; Madgwick et al., 2006). Through the Mos-Erk pathway, Emi2 protein is stabilized and suppresses APC, which leads to MII arrest. Expression of cell cycle regulators, such as cyclin B, Mos and Emi2, is dependent on cytoplasmic polyadenylation-mediated translational activation (Gebauer and Richter, 1997; Barkoff et al., 2000; Han et al., 2017; Takei et al., 2021).

Poly(A) tail length is regulated by competing activities of poly(A) polymerases and deadenylases in the cytoplasm. In *Xenopus* oocytes, poly(A) ribonuclease (Parn) exerts deadenylase activity on maternal mRNAs (Kim and Richter, 2006); however, it has only a minor, if any, role in mRNA deadenylation in mammalian cells (Yamashita et al., 2005). In mammalian cells, two enzyme complexes that regulate mRNA poly(A) tail length have been identified: the poly(A)-specific nuclease (PAN) complex, which consists of a Pan2 catalytic subunit and a Pan3 accessory subunit, and the CCR4–NOT complex (Yamashita et al., 2005; Parker and Song, 2004).

In mammals, the CCR4–NOT complex is the major deadenylase. It is composed of at least eight subunits: a central scaffold subunit, Cnot1; regulatory subunits, Cnot2, Cnot3, Cnot9, Cnot10, Cnot11; and a deadenylase core consisting of Cnot6 or Cnot6l and Cnot7 or Cnot8 (Collart, 2016; Shirai et al., 2014). As the catalytic core, the CCR4–NOT complex contains two types of subunits. Both subunits possess 3′-5′ poly(A) specific exoribonuclease activity and shorten mRNA poly(A) tails. Cnot7 and Cnot8 are DEDD-type deadenylases, named for the conserved Asp-Glu-Asp-Asp catalytic domain, and they associate with the complex via Cnot1 (Bai et al., 1999; Clark et al., 2004; Viswanathan et al., 2004). In contrast, Cnot6 and Cnot6l are EEP-type deadenylases containing an endonuclease-exonuclease-phosphatase (EEP) domain. They associate with the complex via Cnot7 or Cnot8 (Draper et al., 1995; Liu et al., 1998; Tucker et al., 2002; Chen et al., 2002; Basquin et al., 2012). One CCR4–NOT complex contains one DEDD-type deadenylase and one EEP-type deadenylase. It has been shown *in vitro* that Cnot7 and Cnot8 deadenylate Pabpc-free A tails whereas Cnot6 and Cnot6l remove Pabpc-bound A tails (Yi et al., 2018). The *in vivo* functions of these two types of deadenylases are also different. Whereas cells lacking both Cnot6 and Cnot6l are viable, cells lacking both Cnot7 and Cnot8 are inviable. *Cnot6l* knockout mice are fertile, but the developmental ability of oocytes is reduced (Horvat et al., 2018; Sha et al., 2018). In contrast, *Cnot7* knockout in mice results in infertility in males (Nakamura et al., 2004; Ogawa et al., 2004).

Accumulating evidence indicates that the CCR4–NOT complex is involved in regulating maternal mRNA metabolism (Ma et al., 2015; Yu et al., 2016; Horvat et al., 2018; Sha et al., 2018). However, previous studies were conducted on mice with knockouts of only accessory or single deadenylase subunits of the CCR4–NOT complex; thus, they had only partially reduced deadenylase activity. Therefore, the functional significance of the complex during oocyte maturation was not fully understood. In this study, we address the functional significance of the CCR4–NOT complex in oocyte maturation by analyzing *Cnot7* and *Cnot8* knockout oocytes.

## RESULTS

### CNOT7&8 KO oocytes stop development at metaphase of meiosis-I

**Fig. 1.**
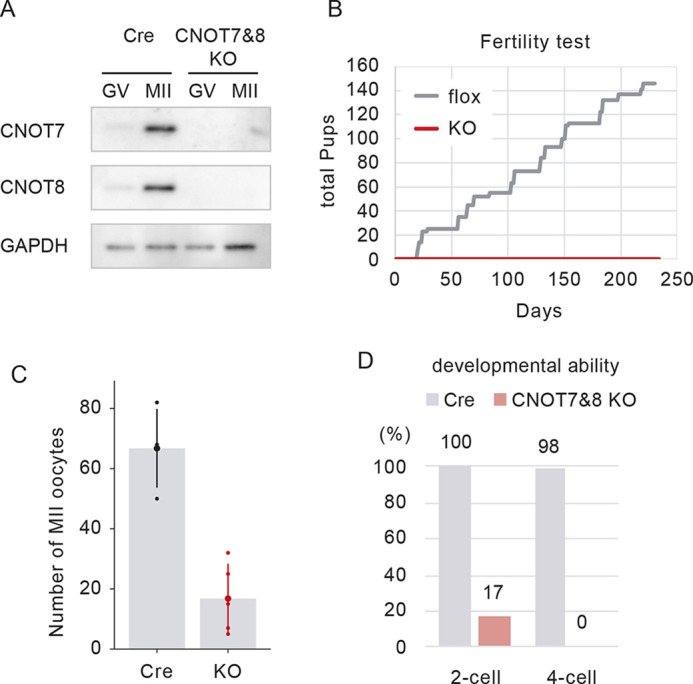
**CNOT7&8 oocytes stop development at an early stage.** (A) Immunoblotting of CNOT7&8 KO oocytes confirming absence of the CNOT7 and CNOT8 in the KO. GAPDH was used as loading control. (B) Fertility test using CNOT7&8 oocyte-specific KO female mice. Female mice were mated with WT male mice. Gray line: total pups from six floxed female to WT male pairs, red line: total pups from six CNOT7&8 oocyte-specific KO female to WT male pairs. (C) The number of oocytes collected after superovulation. Bars and large circle indicate average values. Each smaller circle indicates the number of oocytes collected from one female mouse. Black: Zp3-Cre control mice; red: CNOT7&8 oocyte-specific KO mice. Error bars represent s.d. (D) Developmental ability of CNOT7&8 KO oocytes. MII oocytes were parthenogenetically activated. Only activated oocytes judged by pronuclear formation were subjected to the observation. The percentage of parthenogenetic embryos that developed to the indicated developmental stages is shown. Numbers above bars indicate percentages of embryos. *n*=53 (Zp3-Cre), 42 (CNOT7&8 KO).

We generated mice with oocyte-specific knockouts of Cnot7 and Cnot8 (CNOT7&8 oocyte-specific KO) and confirmed that Cnot7 and Cnot8 levels were depleted in oocytes (Fig. 1A, Fig. S1A). Affected mice were sterile (Fig. 1B). Because we mated CNOT7&8 oocyte-specific KO females with wild-type (WT) males, fertilized cells have *Cnot7* and *Cnot8* genes in a paternal genome and can potentially express them after genome activation at the two-cell stage. Therefore, we expected that embryos from CNOT7&8 KO oocytes might stop development at an early stage of embryogenesis. To test this possibility, we analyzed the *in vitro* development of CNOT7&8 KO oocytes after parthenogenetic activation. The number of CNOT7&8 KO oocytes collected after superovulation was small compared with that of controls (66.8 oocytes in controls, and 16.8 oocytes in CNOT7&8 KO) (Fig. 1C). To analyze developmental activity, MII oocytes were parthenogenetically activated and their development was observed. About 80% of the embryos did not develop to the two-cell stage, and no embryos developed to the four-cell stage (Fig. 1D). These results indicate that CNOT7&8 KO oocytes stop development at the one-cell stage.

**Fig. 2.**
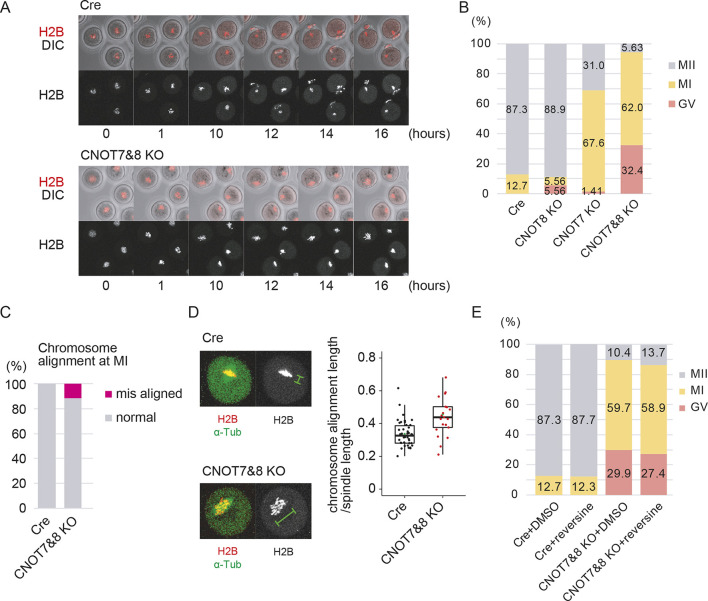
**CNOT7&8 KO oocytes show developmental arrest at metaphase I.** (A) Representative time-lapse images of CNOT7&8 KO oocytes after release from the GV stage. Chromosomes were visualized with H2B-mRFP. Time after germinal vesicle breakdown (h) is shown. (B) Developmental ability of CNOT7&8 KO oocytes after release from the GV stage. Percentage of oocytes reaching each developmental stage 20 h after release from the GV stage are shown. *n*=79 (Zp3-Cre), 36(CNOT8 KO), 142 (CNOT7 KO), 71 (CNOT7&8 KO). (C) Chromosome alignment at the MI spindle. Live-cell images of H2B-mRFP after release from the GV stage were analyzed. *n*=44 (Zp3-Cre), 52 (CNOT7&8 KO). (D) Representative images and measurement of chromosome alignment of CNOT7&8 KO oocytes in MI spindle. Green brackets indicate chromosome alignment length. The plot indicates chromosome alignment length divided by spindle length. Each dot indicates a spindle. Vertical lines indicate the range of the data. Upper, middle and lower horizontal lines indicate upper quartile, median and lower quartile, respectively. *n*=42 (Zp3-Cre), 21 (CNOT7&8 KO). *P=*1.2×10^−3^ (Student's *t*-test). (E) Effect of reversine on MI arrest in CNOT7&8 KO oocytes. CNOT7&8 KO oocytes were released from the GV stage in the presence of 5 µM reversine. Percentage of oocytes reaching each developmental stage 20 h after release from the GV stage are shown. *n*=55 (Zp3-Cre+DMSO), 73 (Zp3-Cre+reversine), 67 (CNOT7&8 KO+DMSO), 95 (CNOT7&8 KO+reversine).

To analyze the developmental capacity of CNOT7&8 KO oocytes more precisely, we performed live-cell oocyte imaging and observed their meiotic maturation. Fully grown germinal vesicle (GV)-stage oocytes were collected from female CNOT7&8 oocyte-specific KO mice 48 h after intraperitoneal injection of pregnant mare serum gonadotropin (PMSG). To visualize chromosomes and microtubules, red fluorescent protein fused to histone H2B (H2B-mRFP) and enhanced green fluorescent protein coupled with α-tubulin (EGFP-α-Tub) were exogenously expressed in these oocytes by mRNA injection. Whereas a large proportion of oocytes with single knockouts of *Cnot7* or *Cnot8* developed to MII, only 5% of CNOT7&8 KO oocytes did so (Fig. 2A,B). Most CNOT7&8 KO oocytes showed developmental arrest at metaphase-I (MI) (Fig. 2B). Although approximately 90% of them showed normal chromosome alignment at MI, a small number had misaligned chromosomes (*P*<0.05, Fisher's exact test) (Fig. 2C). In addition, chromosomes were slightly dispersed on the MI spindle in CNOT7&8 KO (Fig. 2D). We next tested whether these errors in MI spindle formation caused MI arrest in CNOT7&8 KO. To test the effect of the spindle assembly checkpoint (SAC), we used reversine, an inhibitor of SAC kinase monopolar spindle 1 (Mps1; Ttk), to inactivate the SAC. We found that CNOT7&8 KO oocytes still arrested in MI (Fig. 2E). These results suggest that, although chromosome alignment in CNOT7&8 KO oocytes showed small abnormalities, the arrest of oocytes in MI cannot be explained solely by SAC activation.

### The CCR4–NOT complex suppresses translational activity of maternal mRNAs

**Fig. 3.**
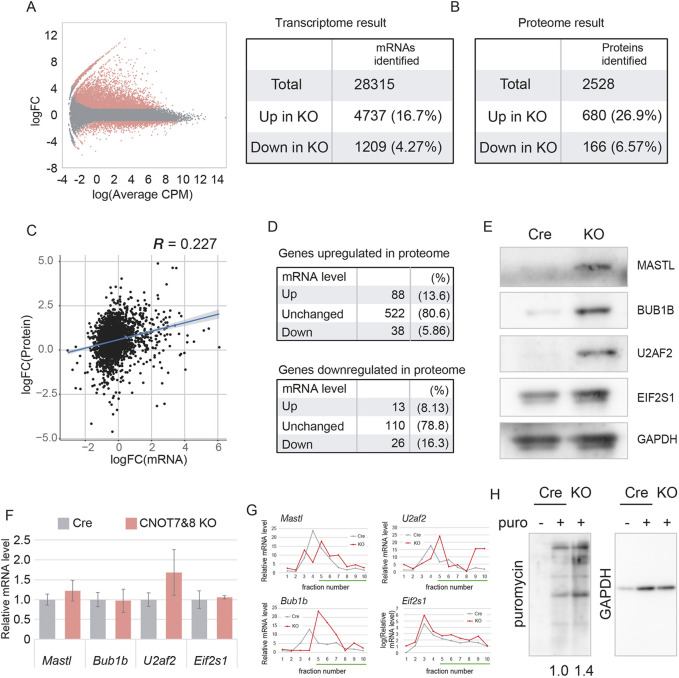
**Gene expression in CNOT7&8 KO oocytes is largely altered owing to translational activation of maternal mRNAs.** (A) Scatter plot of changes of mRNA expression between Cre control and CNOT7&8 KO GV oocytes. Genes for which expression was decreased or increased in CNOT7&8 KO more than 2-fold are colored red. The table summarizes the total number of genes and the number of DEGs identified. (B) Summary of mass spectrometry analysis of Cre control and CNOT7&8 KO GV oocytes. The number of proteins for which the abundance ratio (KO/Cre) increased or decreased more than 2-fold is shown. (C) Scatter plot comparing changes detected by transcriptomic and proteomic analyses. Each dot indicates logged fold-change value (KO/Cre) of each gene. The blue line and gray area indicate the regression line and its 95% confidence interval. *R* indicates the correlation coefficient. (D) Summary of the analysis in C. (E) Immunoblotting showing upregulated proteins. Lysates of 30 oocytes were loaded in each lane. Western blotting was performed for the indicated genes. Experiments were repeated at least twice, and representative images are shown. GAPDH was used as loading control. (F) Expression levels of mRNAs of the proteins shown in E. mRNA levels were quantified by reverse transcription followed by quantitative PCR (RT-qPCR). Bars indicate mean expression values relative to spike-in RNA. Error bars indicate s.e.m. *n*=3. No genes showed a significant difference (*P*>0.05, Student's *t*-test). (G) Distribution of mRNAs on polysome profile. Polysome fractionation by sucrose density gradient centrifugation followed by RT-qPCR was performed. Values relative to Cre fraction 1 are shown. Green lines indicate polysome fractions. (H) Immunoblotting of puromycylated peptides. Thirty oocytes were cultured with 5 µg/ml puromycin for 30 min and then analyzed by western blotting. Numbers below the blot indicate mean relative signal intensity of two experiments. GAPDH was used as loading control.

We analyzed the effects of *Cnot7* and *Cnot8* deletions on oocyte transcriptomes and proteomes. Total RNA was extracted from CNOT7&8 KO oocytes at the GV stage, and mRNAs were purified by ribosomal RNA depletion and subjected to RNA-sequencing (RNA-seq) analysis. Transcriptomic data showed that a relatively large number of transcripts were upregulated in CNOT7&8 KO oocytes (4737 upregulated versus 1209 downregulated; Fig. 3A). As reported in several studies (Ma et al., 2015; Horvat et al., 2018; Sha et al., 2018), the CCR4–NOT complex destabilizes maternal transcripts. Our data suggest that the deadenylases Cnot7 and Cnot8 are equally important for regulation of maternal mRNA metabolism. Next, to acquire shotgun proteomic data, nanoscale liquid chromatography coupled to tandem mass spectrometry (nanoLC-MS/MS) was performed on the oocytes, identifying more than 2500 proteins (680 upregulated versus 160 downregulated, fold change<2.0; Fig. 3B). We then compared and analyzed the correlation between transcriptomic and proteomic data, finding only a weak, if any, correlation between them (R=0.23; Fig. 3C). Importantly, a large majority of genes with upregulated protein levels had unchanged or decreased mRNA levels (86%; Fig. 3D), suggesting that, for these genes, upregulation of protein expression was not due to an increase of their transcripts. To validate this, we tested the translational activities of genes with upregulated protein, but unchanged mRNA levels in CNOT7&8 KO oocytes. Those related to the cell cycle (Mastl), spindle formation (Bub1b), splicing (U2af2), and translation initiation (Eif2s1) were upregulated at the protein level, but not at the mRNA level (Fig. 3E,F). Then, we tested translational activities of these genes by polysome fractionation and found that their distribution was shifted to heavier polysome fractions in CNOT7&8 KO oocytes (Fig. 3G). To validate this, we performed a puromycin protein synthesis assay in which translational activity was examined by measuring puromycin incorporation (Bergeman and Huot, 2017). After treatment with puromycin for 30 min, puromycylated peptides were increased at the GV stage of CNOT7&8 KO oocytes, compared with control oocytes, indicating upregulation of translational activity in the absence of Cnot7 and Cnot8 (Fig. 3H).

### The CCR4–NOT complex suppresses translation of CPE-containing mRNAs

**Fig. 4.**
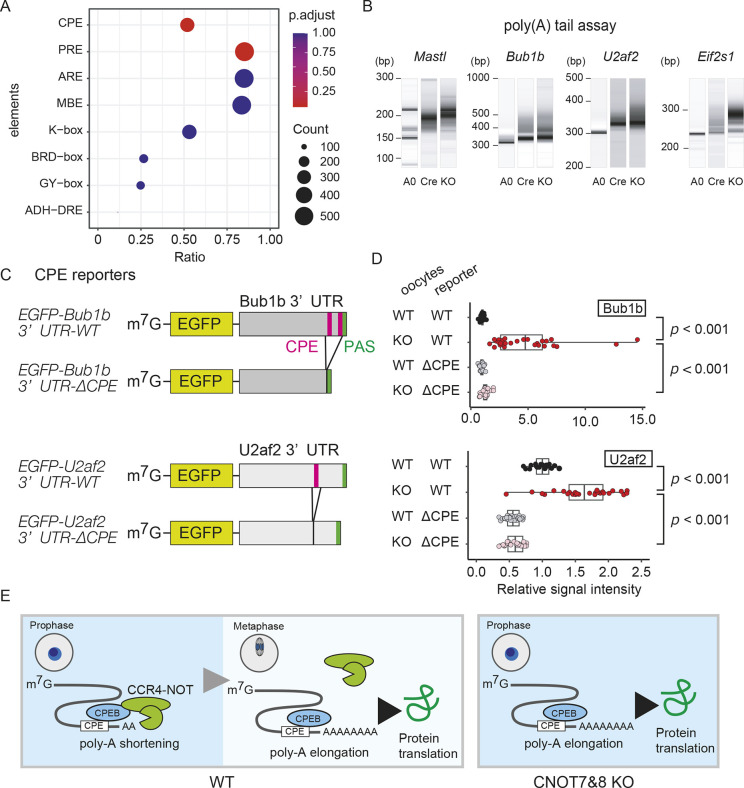
**Genes with CPE in their 3′UTRs are translationally activated in CNOT7&8 KO oocytes.** (A) Enrichment analysis of cis-regulatory elements on mRNA 3′UTRs of upregulated proteins in CNOT7&8 KO oocytes. mRNA 3′UTRs of genes for which protein expression was upregulated were subjected to enrichment analysis of cis-regulatory elements. The dot plot indicates the number of genes with each cis-regulatory element and its ratio. Adjusted *P*-value (chi-squared test); CPE: 0.053; PRE: 0.062; others: 1.0. (B) Poly(A) tail assay on the translationally activated mRNAs. Poly(A) tail lengths were analyzed by poly(A) tail assay on indicated genes. A0, amplicon from poly(A) removed samples. (C) Design of CPE reporter mRNAs. Magenta and green regions indicate CPE and polyadenylation signal (PAS), respectively. (D) CPE reporter assay in CNOT7&8 KO GV oocytes. CNOT7&8 KO or WT GV oocytes were injected with EGFP-Bub1b or U2af2-3′UTR-WT or ΔCPE. Each circle indicates relative EGFP signal intensity. Horizontal lines indicate data range. Left, middle and right vertical lines indicate lower quartile, median and upper quartile, respectively. Student's *t*-test. (E) Graphical summary of the proposed regulatory mechanism of translational activity of maternal mRNAs by the CCR4–NOT complex. The CCR4-NOT complex targets CPE-containing mRNAs to suppress their untimely translational activation by shortening their poly(A) tails.

To investigate the mechanism by which Cnot7 and Cnot8 regulate translational activity, we performed motif analysis on 3′UTRs of genes upregulated in the CNOT7&8 KO oocyte proteome and found that the CPE motif sequence is enriched in these 3′UTRs (Fig. 4A). CPEs serve as binding sites for CPEB, which can bind to the CCR4–NOT complex (Ogami et al., 2014). Genes for which translational activation was confirmed in this study (Fig. 3G) have consensus CPE sequences in their 3′UTRs and have elongated poly(A) tails in CNOT7&8 KO oocytes (Fig. 4B). To test whether the CPE sequence is related to translational upregulation in CNOT7&8 KO oocytes, we employed a reporter assay in which mRNA of the EGFP open-reading frame with either WT *Bub1b* or *U2af2* 3′UTR or lacking CPE was injected into CNOT7&8 KO oocytes. For injection control, we injected RFP mRNA with poly(A) sequence and EGFP signal was normalized to the RFP signal. The expression level of EGFP was higher in the WT *Bub1b* or *U2af2* 3′UTR construct than in the construct lacking CPE (Fig. 4C,D). We also tested EGFP expression with these constructs in control oocytes and found that the expression level of EGFP was lower in control oocytes than in CNOT7&8 KO oocytes in the WT construct but not in the construct lacking CPE (Fig. 4D). These results suggest that the CCR4–NOT complex may target the CPE sequence to suppress translational activation of CPE-containing mRNAs by shortening their poly(A) tails.

Because a previous study with *Cnot6l* knockout oocytes showed upregulation of ribosomal protein mRNAs (Sha et al., 2018) in *Cnot6l* KO GV oocytes (Sha et al., 2018), we analyzed the expression of ribosomal proteins in CNOT7&8 KO oocytes based on the transcriptomic and proteomic data. Expression of ribosomal proteins or their mRNAs was mostly unchanged (protein: 92%; mRNA: 89%), with only a small fraction showing upregulation (protein: 2.0%; mRNA: 7.8%; Fig. S2). However, gene ontology (GO) analysis of genes upregulated in the CNOT7&8 KO oocyte proteome showed that translation initiation-related genes were enriched (Fig. S3). These results suggest that upregulation of translation-related genes may also alter translational activity in CNOT7&8 KO oocytes.

### Untimely Emi2 translational activation may be involved in meiosis I arrest in CNOT7&8 KO oocytes

During oocyte maturation, translation of CPE-containing mRNAs is activated after meiotic resumption. Our results so far suggest that this system is defective in CNOT7&8 KO oocytes, resulting in untimely induction of translational activation. In normal oocytes, timely translational activation is a key mechanism for arresting the oocyte cell cycle at MII. We hypothesized that untimely activation of the MII arrest system might induce developmental arrest at MI in CNOT7&8 KO oocytes. Therefore, we investigated Emi2 expression, which is central to the MII arrest system. In normal oocytes, expression of Emi2 is restricted to MII (Shoji et al., 2006; Madgwick et al., 2006). Interestingly, we found that Emi2 is expressed at MI in CNOT7&8 KO oocytes (Fig. S4A). We then tested whether MI arrest in CNOT7&8 KO oocytes is caused by untimely Emi2 expression. When we destabilized Emi2 by inhibiting Erk activity with the mitogen-activated protein/ERK kinase (Mek) inhibitor trametinib, the ratio of CNOT7&8 KO oocytes that were released from MI increased (Fig. S4B). Although injection of siRNAs for Emi2 alone had little effect on release from MI arrest in CNOT7&8 KO oocytes, injection of siRNAs for Emi2 together with reversine increased the ratio of CNOT7&8 KO oocytes that were released from MI (Fig. S4C). These results suggest that, although it is not a sole factor, untimely Emi2 expression, together with chromosome alignment errors, is possibly involved in MI arrest in CNOT7&8 KO oocytes.

To address how untimely EMI2 expression is induced by loss of Cnot7 and Cnot8, we analyzed the poly(A) tail lengths of *Emi2* mRNAs and found that they were elongated in MI to almost the same length as in MII controls (Fig. S4D). It is possible that poly(A) tail elongation stabilizes *Emi2* mRNAs, perhaps leading to their overexpression. However, the amount of *Emi2* mRNA was unchanged between GV-stage control and MI (Fig. S4E). Because cytoplasmic poly(A) tail elongation activates translation of *Emi2* mRNAs in MII oocytes (Takei et al., 2021), we tested the translational activity of *Emi2* mRNAs in CNOT7&8 KO oocytes. Polysome fractionation by sucrose density ultracentrifugation and qPCR showed that the distribution of *Emi2* mRNA was shifted to heavier polysomes in CNOT7&8 KO oocytes (Fig. S4F), indicating upregulation of *Emi2* mRNA translational activity. Additionally, EGFP expression of the reporter mRNA with *Emi2* 3′UTR was higher in CNOT7&8 KO (Fig. S4G,H). In contrast to *Bub1b* or *U2af2* 3′UTR reporter constructs, the *Emi2* 3′UTR reporter showed no significant difference in EGFP expression between the WT construct and the construct lacking CPE, suggesting that translational upregulation of Emi2 in CNOT7&8 cannot be explained only by CPE (Fig. S4G,H). Taken together, these results suggest that Cnot7 and Cnot8 loss in oocytes induces untimely elongation of *Emi2* mRNA poly(A) tails at MI, thereby activating Emi2 translation, and that untimely Emi2 expression is possibly involved in MI arrest.

## DISCUSSION

This study provides clear evidence that the CCR4–NOT complex regulates maternal mRNA translational activity through regulation of poly(A) tail lengths. The CCR4–NOT complex is involved in maternal mRNA degradation. This physiological function was deduced from analyses of mice with knockouts of accessory or single deadenylase subunits of the complex. Analysis of *Cnot6l* knockout mice revealed that Cnot6l regulates maternal mRNA decay (Horvat et al., 2018; Sha et al., 2018), but the effect is limited to a subset of transcripts, probably because Cnot7 and Cnot8 deadenylate a large percentage of transcripts. Here, we show that knockout of both *Cnot7* and *Cnot8* in oocytes resulted in drastic changes in both transcriptome and proteome before meiotic resumption. Protein expression of Cnot7 and Cnot8 is low at the GV stage, but it increases after resumption of meiosis (Fig. 1C). Our data show that, despite relatively low expression, Cnot7 and Cnot8 are required for regulation of gene expression at the GV stage. Given that Cnot6 and Cnot6l cannot associate with the complex without Cnot7 or Cnot8, deadenylase activity associated with the complex is completely abolished in our knockout system despite free Cnot6 or Cnot6l in the cells. Each catalytic subunit of the CCR4–NOT complex has its own targets, but they largely overlap. As a result of this functional redundancy, depletion of a single catalytic subunit may be not sufficient to detect effects of altered translational activity. This may explain why translational control by the CCR4–NOT complex was not addressed in previous studies.

We found that CNOT7&8 KO oocytes arrest the cell cycle at MI and that untimely expression of Emi2 is possibly involved in MI arrest. In addition to untimely Emi2 expression, spindle formation errors also contribute to MI arrest. Several studies have shown that depletion of CCR4–NOT complex subunit proteins causes spindle formation errors (Pasternak et al., 2016; Sha et al., 2018). Just as these studies could not specify the factors that cause spindle formation error, we also cannot, because expression of so many genes was altered in CNOT7&8 KO oocytes. As for the CSF pathway, we identified Emi2 as one of the possible responsible factors for MI arrest in CNOT7&8 KO. Because Emi2 is the factor farthest downstream in the CSF pathway, and because Emi2 expression is regulated by translational control via cytoplasmic polyadenylation, we only tested Emi2 in this study, but it is possible that other upstream factors in the CSF pathway are also involved in MI arrest in CNOT7&8 KO oocytes.

In this study, we showed that poly(A) tail shortening by the CCR4–NOT complex is crucial for suppressing untimely translational activation of maternal mRNAs. We provide evidence that translational control by the CCR4–NOT complex is mediated by CPE in several maternal mRNAs. Translational regulation by CPE is well studied in *Xenopus* oocytes and early-stage embryos. In *Xenopus* oocytes, Parn suppresses translational activation of maternal mRNAs by deadenylating them before resumption of meiosis. Before resumption of meiosis, CPEB recruits Parn to CPE-containing mRNAs, which facilitates deadenylation and maintains a dormant state (Kim and Richter, 2006). When oocytes are stimulated by progesterone, CPEB is phosphorylated by mitotic kinases such as aurora kinase A, which leads to expulsion of Parn (Sarkissian et al., 2004). CPEB also serves as a docking site for a polyadenylation complex to elongate poly(A) tails and to activate translation after Parn is released from the ribonuclear complex (Barnard et al., 2004; Kim and Richter, 2006). Therefore, in *Xenopus* oocytes, CPE functions as a switch that changes maternal mRNAs from deadenylated and translationally dormant to polyadenylated and translationally active, during oocyte maturation. In mammals, Parn is less important for regulation of poly(A) tails (Yamashita et al., 2005), and the deadenylase that is responsible for the regulation of poly(A) tails of CPE-containing mRNAs was formerly unknown. Here, we propose that the CCR4–NOT complex is the deadenylase that switches off translational activity of CPE-containing mRNAs during mammalian oocyte maturation. We propose that mammalian oocytes utilize the CCR4–NOT complex to equip a conserved system that prevents untimely translational activation of maternal mRNAs.

We found that various genes have altered protein expression in CNOT7&8 KO oocytes independently of the CPE. Some genes showed correlations between increases in mRNA and protein levels, which implies that upregulation of protein expression in these genes is mediated by stabilization and accumulation of transcripts. We also found that genes without CPEs in their 3′UTRs showed increased protein expression, despite their unchanged mRNA levels. Upregulation of expression of these genes could be due to stabilization of proteins or translational activation of their mRNAs. One possible explanation is that increased expression of translation initiation-related genes may upregulate translation of mRNAs without CPE. Our GO analysis of increased proteins in CNOT7&8 KO oocytes showed enrichment of translation initiation-related genes (Fig. S3). Similarly, another group showed that *Cnot6l* KO upregulates expression of ribosomal protein genes (Sha et al., 2018). Another possibility is that the CCR4–NOT complex may also regulate translation of mRNAs having cis-regulatory elements other than CPE. Previous studies have identified several sequence elements for recruitment of the CCR4–NOT complex, such as the AU-rich element (ARE) and the Pumilio response element (PRE) (Lykke-Andersen and Wagner, 2005; Goldstrohm et al., 2006; Vieux and Clarke, 2018). Our cis-regulatory element search also showed that these elements are found in a large number of upregulated genes (Fig. 4A). Future studies will clarify involvement of the CCR4–NOT complex in translational control of mRNAs having these elements. Our CNOT7&8 KO oocyte system provides a powerful tool to address the physiological importance of these elements.

## MATERIALS AND METHODS

### Mice and oocytes

*Cnot7* conditional KO (*Cnot7^fl/fl^*) mice (accession number CDB0036E) and *Cnot8* conditional KO (*Cnot8^fl/fl^*) mice (accession number CDB0584K) were generated with TT2 embryonic stem cell (ES cell) lines (Yagi et al., 1993). Targeted ES cells were injected into 8-cell stage embryos of ICR mice to generate chimeric mice. Chimeric mice were crossed with C57BL/6J mice to generate F1 heterozygotes. To generate conditional alleles (floxed alleles) from targeted alleles, mice with targeted alleles were crossed with mice expressing FLP (#009086; The Jackson Laboratory). The absence of FLP knock-in alleles in mice with floxed alleles was confirmed by PCR. We backcrossed *Cnot7^fl/fl^* and *Cnot8^fl/fl^* mice with C57BL/6J mice for at least eight generations. To generate conditional knockout alleles, *Cnot7^fl/fl^* and *Cnot8^fl/fl^* mice were crossed with Zp3-Cre mice (#003394; The Jackson Laboratory).

All mice were group housed under specific pathogen-free (SPF) conditions with a 12-h day/night cycle environment with chow diet and water available *ad libitum*. Females having a C57BL/6J background (4 weeks old) were superovulated with intraperitoneal injections of 5 IU PMSG and 5 IU human chronic gonadotropin (hCG) at 48-h intervals. GV oocytes were collected 48 h post-PMSG injection. MII oocytes were collected 16 h post-hCG injection. Cumulus cells were removed by exposure to M2 medium (Sigma-Aldrich) containing 100 μg/ml hyaluronidase for <5 min, followed by pipetting into fresh M2 medium. GV oocytes were cultured in M16 medium (Sigma-Aldrich) supplemented with 500 μM dibutyryl-cAMP (dbcAMP) (Sigma-Aldrich) at 37°C under 5% CO_2_ until use. MII oocytes and fertilized embryos were cultured in M16 medium at 37°C under 5% CO_2_. Experiments with animals were carried out in accordance with guidelines for animal use issued by the Committee for Animal Experiments in Okinawa Institute of Science and Technology Graduate University.

### Live-cell imaging

mRNAs were synthesized *in vitro* with linearized template plasmids using a RiboMax^TM^ Large Scale RNA Production System-T7 (Promega) supplemented with Ribo m^7^G Cap Analog (Promega) in reaction mixtures. Reaction mixtures were treated with DNase I after RNA synthesis. Synthesized RNA was purified with phenol extraction followed by ethanol precipitation (Yamashita et al., 2005). mRNAs (10 ng/μl) were injected into GV oocytes with a Piezo-driven micromanipulator (Prime Tech) and cultured for 1 h before being released from the GV stage by transferring into dbc-AMP-free M16 medium. mRNA-injected oocytes were transferred to medium drop-covered with mineral oil in a glass-bottomed dish and observed with a Leica TCS SP8 LIGHTNING confocal microscope equipped with a motorized stage, LAS X software and a CO_2_ microscope stage incubator. Samples were scanned from bottom to top (number of optical sections: 18-21; optical section spacing: 3 μm; imaging medium: M16). For time lapse imaging, samples were scanned from bottom to top (number of optical sections: 10; optical section spacing: 6 μm; imaging medium: M16) at 30-min intervals. For EGFP signal intensity measurement, image stacks were quick-projected and analyzed with ImageJ software (NIH).

### *Emi2* knockdown in oocytes

Control siRNAs or *Emi2* siRNA (20 μM) and mRNAs of H2B-mRFP were injected into GV oocytes and cultured for 1 h before being transferred into dbcAMP-free M16. Oocytes were cultured 18 h after the medium change and subjected to live imaging. Live-cell imaging was performed under the conditions described above.

### Total RNA-seq

Total RNA was isolated from GV oocytes using TRIzol reagent (Invitrogen). One-hundred oocytes were used for RNA-seq library preparation with a TruSeq Stranded mRNA LT Sample Prep Kit (Illumina), which allows ribosomal RNA depletion-based purification of mRNA, according to the manufacturer's protocol with 11 PCR cycles. 150-bp, paired-end read RNA-seq was performed with a NovaSeq 6000 SP flow cell (Illumina). For data analysis, using nf-core/rnaseq pipeline v2.0 (Ewels et al., 2020), reads were mapped to the GRCm38 genome database using STAR aligner (v2.6.1d) (Dobin et al., 2013). Read counts were normalized using the package EdgeR (v3.11) (Robinson et al., 2010) and a cut-off of at least 0.2 counts per million (CPM) in two samples was selected. Differentially expressed genes (DEGs) were statistically tested using EdgeR's exact test, and genes with false discovery rate ≤0.05, *P*≤0.05 and fold change ≥2 or ≤−2 were considered as DEGs.

### Mass spectrometry analysis

One-hundred oocytes were lysed in 8 M urea. The proteins were reduced with 1 mM dithiothreitol for 90 min and alkylated with 5.5 mM iodoacetamide for 30 min. After digestion with lysyl endopeptidase, mass spectrometry grade (Fuji Film Wako Chemicals) at 37°C for 3 h; the resulting peptide mixtures were diluted with 10 mM Tris-HCl (pH 8.2) to achieve a final concentration below 2 M urea and subsequently digested with Trypsin Gold, mass spectrometry grade (Promega) at 37°C for 3 h. An equal amount of trypsin was then added once more for overnight digestion and the fragmented peptides were desalted using ZipTip C18 (Millipore). Shotgun proteomic analyses were performed using an Orbitrap Eclipse Tribrid mass spectrometer with FAIMS Pro interface (Thermo Fisher Scientific), which was connected to the Ultimate 3000 RSLCnano system (Thermo Fisher Scientific). The peptide samples were applied to a 3 µm C18 NANO HPLC CAPILLARY COLUMN75-3-10 (Nikkyo Technos) and separated using a linear gradient of 2-24% mobile phase (0.1% formic acid in acetonitrile) at 300 nl/min. Full-scan MS spectra were acquired with a resolution of 120,000 and subsequent MS/MS scans were performed in the ion trap using higher energy collisional dissociation (HCD) fragmentation with a normalized collision energy of 35% with 10 ms maximum injection time. Protein identification was conducted by searching against the UniProt mouse reference proteome database (UP000000589) using Sequest HT algorithm in Proteome Discoverer Software (version 2.5) (Thermo Fisher Scientific) based on the statistical criterion of a false discovery rate <1%.

### Cis-regulatory element enrichment analysis

Enrichment of cis-regulatory elements were analyzed on 3′UTRs of the genes for which expression levels were upregulated in proteome analysis. The sequences of 3′UTR of the genes were obtained from BioMart (Ensembl). For motif sequences, UUUUAU (CPE), UGUAAAUA (PRE), AUUUA (ARE), AUAGU (MBE), UGUGAU (K-box), AGCUUUA (BRD-box), GUCUUCC (GY-box) and AAGGCUGA (ADH-DRE) were used. Enrichment of each motif at the 3′UTR of the genes upregulated in the proteome was compared with those that were unchanged or downregulated, assessed by chi-squared test and Bonferroni-corrected *P*-values were calculated.

### Quantitative real-time PCR after sucrose density gradient ultra-centrifugation

GV oocytes were lysed in polysome extraction buffer (15 mM Tris-HCl pH 7.4, 300 mM NaCl, 15 mM MgCl_2_, 1% Triton X-100, 1 mg/ml heparin, 100 μg/ml cycloheximide, and protease inhibitors) and nuclei were removed by centrifugation (14,000 ***g*** for 5 min at 4°C). Cell lysates were loaded onto a linear sucrose density gradient (5–50%). After ultracentrifugation with an SW41Ti rotor (Beckman Coulter) at 35,000 rpm (250,000 ***g***) for 200 min, fractions were collected and RNA was extracted using IsogenII reagent (Nippon Gene). cDNA was generated with total RNA, random primers (Thermo Fisher Scientific), and SuperScript Reverse Transcriptase III (Thermo Fisher Scientific). cDNA was mixed with primers and SYBR Green Supermix (Takara Bio) and analyzed with a Viia 7 sequence detection system (Applied Biosystems). Relative mRNA expression was determined after normalization with control mRNA levels of the first fraction using the ΔΔCt method. Primers are listed in Table S1.

### Immunoblotting

Thirty oocytes were lysed in SDS sample buffer (2% SDS, 0.125 M Tris, 0.2% 2-mercaptoethanol, 10% glycerol, 0.01% Bromophenol Blue). Lysates were subjected to SDS-polyacrylamide gel electrophoresis followed by electro-transfer onto Immobilon-P membranes (Millipore). Protein bands were blotted with primary antibodies and ECL anti-rabbit or mouse IgG HRP-linked whole antibody (GE Healthcare) as the secondary antibody. For detection, we used Immobilon Western HRP substrate (Millipore). To quantify the results, we used ImageQuant software in an Image Analyzer LAS 4000 mini (GE Healthcare) or Amersham Imager 680 (GE Healthcare).

### Poly(A) tail assay

Poly(A) tail lengths of mRNAs were analyzed using Poly(A) Tail-Length Assay Kits (Affymetrix), according to the manufacturer's protocol. Briefly, total RNA was incubated with poly(A) polymerase in the presence of guanosine (G) and inosine (I) to add a GI tail at the 3′-ends of poly(A)-containing RNAs. cDNA was generated with PAT universal primer and reverse transcriptase using GI-tailed RNA as a template. PCR amplification was performed with gene-specific and PAT universal primers and HotStart-IT Taq DNA polymerase. For detection of non-adenylated fragments, total RNA was mixed with 5 μM of the oligo (dT) primer (TTTTTVN; Invitrogen) and treated with 0.2 U of RNase H (Invitrogen) at 37°C for 30 min, which degrades the RNA strand of RNA–DNA hybrids. Processed RNAs were then subjected to adaptor ligation, reverse transcription, and PCR reaction. Primers used in these experiments are listed in Table S1. PCR products were analyzed with a BioAnalyzer (Agilent).

### Quantification and statistical analysis

Statistical analyses were performed using unpaired, two-tailed Student's *t*-test, and chi-squared test. Statistical parameters are shown in figure legends. For all representative images, experiments were reproduced more than two times. All experiments with oocytes were repeated two or more times.

## Supplementary Material

10.1242/develop.201773_sup1 Supplementary information

Table S1. Primer list
