# Supplementary material for: The CCR4–NOT complex suppresses untimely translational activation of maternal mRNAs
Source: Development. 2023 Oct 18;150(21):dev201773. doi: 10.1242/dev.201773 (PMC10617601; doi:10.1242/dev.201773)
Supplement: Supplementary information [file develop-150-201773-s1.pdf]

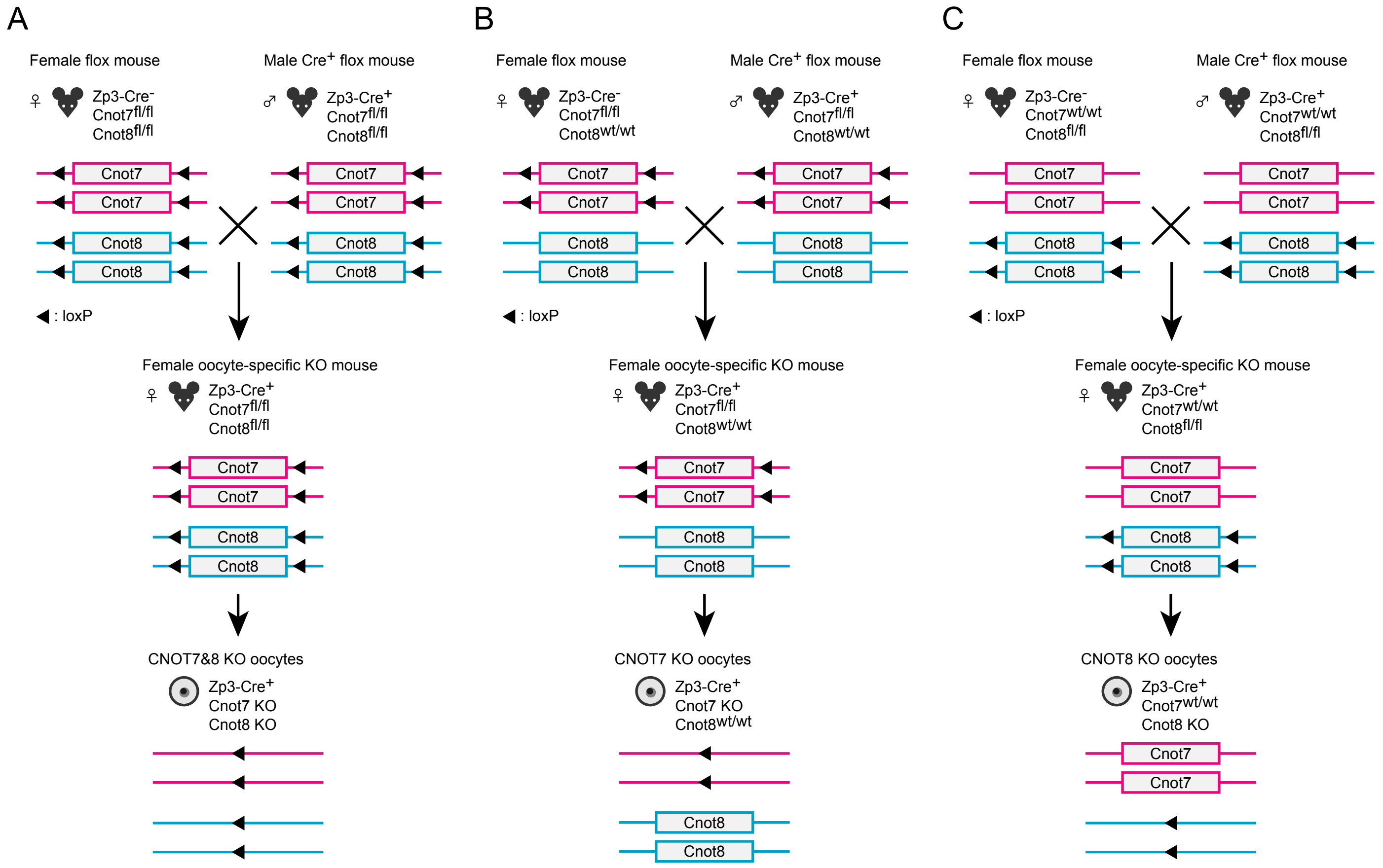

Fig. S1. Strategy for generation of conditional knockout mice. (A, B, C) Strategy for genration of mice with oocyte-specific knockout of both Cnot7 and Cnot8 (A), Cnot7 alone (B), and Cnot8 alone (C).

Development • Supplementary information

A

Name	RNA-seq FC	proteome FC
Rpl10l	6.16	#N/A
Rpl9	3.82	#N/A
Rpl3l	3.30	#N/A
Rpl24	2.27	1.46
Rpl7l1	2.13	#N/A
Rpl3	1.26	1.23
Rpl7	1.21	1.17
Rpl23a	1.06	1.55
Rpl36a	1.05	#N/A
Rpl10	1.01	0.94
Rpl34-ps1	1.01	#N/A
Rpl15	0.90	1.28
Rpl37a	0.87	1.52
Rpl23	0.85	#N/A
Rpl6	0.84	1.18
Rpl39	0.84	2.20
Rpl41	0.82	#N/A
Rpl39l	0.80	#N/A
Rplp0	0.79	0.89
Rpl27	0.78	#N/A
Rpl11	0.75	1.16
Rpl21	0.75	#N/A
Rpl31	0.75	1.29
Rpl34	0.74	#N/A
Rpl4	0.74	1.23
Rpl27a	0.73	1.38
Rpl26	0.72	1.67
Rpl7a	0.72	1.23
Rpl35a	0.71	#N/A
Rpl38	0.70	#N/A
Rpl32	0.70	#N/A
Rpl13	0.70	1.43
Rpl14	0.70	1.00
Rpl12	0.69	0.97
Rpl22	0.69	#N/A
Rplp2	0.68	1.59
Rpl35	0.67	1.62
Rpl5	0.67	1.39
Rpl8	0.66	1.46
Rpl17	0.65	1.15
Rpl37	0.64	#N/A
Rpl36al	0.64	#N/A
Rpl10a	0.62	#N/A
Rpl30	0.62	#N/A
Rpl22l1	0.60	#N/A
Rpl28	0.60	1.30
Rpl13a	0.60	#N/A
Rpl18a	0.59	1.08
Rpl29	0.57	#N/A
Rpl18	0.56	0.90
Rplp1	0.53	0.43
Rpl19	0.49	#N/A
Rpl37rt	0.42	#N/A
Rpl36	0.39	1.12

Name	RNA-seq FC	proteome FC
Rps6ka4	6.90	#N/A
Rps19-ps3	2.94	#N/A
Rps23	2.77	0.98
Rps3a2	1.63	#N/A
Rps3a3	1.42	#N/A
Rps6ka2	1.21	#N/A
Rps27	1.20	#N/A
Rps3a1	1.18	#N/A
Rps6ka6	1.13	#N/A
Rps6ka5	1.07	#N/A
Rps6kb1	1.03	#N/A
Rps3	1.02	0.77
Rps27rt	1.00	#N/A
Rps6kc1	0.90	#N/A
Rps4l	0.88	#N/A
Rps19bp1	0.87	#N/A
Rps6kb2	0.87	#N/A
Rps4x	0.86	1.10
Rps27a	0.86	#N/A
Rps27l	0.85	#N/A
Rps18	0.85	0.97
Rps20	0.84	1.22
Rps29	0.82	0.40
Rps25	0.79	1.29
Rps6ka1	0.77	#N/A
Rps15	0.76	1.43
Rps6	0.76	#N/A
Rps24	0.75	#N/A
Rps15a	0.75	0.33
Rps14	0.75	1.27
Rps13	0.75	1.05
Rps26	0.73	#N/A
Rps16	0.71	#N/A
Rps19	0.71	1.64
Rps11	0.69	1.39
Rps28	0.69	1.41
Rps12	0.68	1.15
Rps7	0.67	1.17
Rps6ka3	0.67	1.85
Rps17	0.65	1.18
Rps2	0.65	#N/A
Rps9	0.65	0.35
Rps8	0.64	#N/A
Rpsa	0.63	#N/A
Rps10	0.58	1.50
Rps21	0.57	1.57
Rps5	0.55	1.20
Rps6kl1	0.54	#N/A
Rps12l1	0.14	#N/A

B

Up (mRNA)	7.77	%
Up (protein)	1.96	%

Down (mRNA)	2.91	%
Down (Protein)	5.88	%

Uncanged (mRNA)	89.32	%
Unchanged (Protein)	92.16	%

Fig. S2. Expressions of boh ribosomal proteins and their mRNAs were not largely affected in CNOT7&8 KO oocytes. (A) Expression levels of ribosomal proteins and their mRNAs. Fold-change value (KO/Cre) calculated from transcriptome and proteome results. Red and blue indicate genes of which expression levels were increased or decreased more than 2 times. (B) Summary of (A).

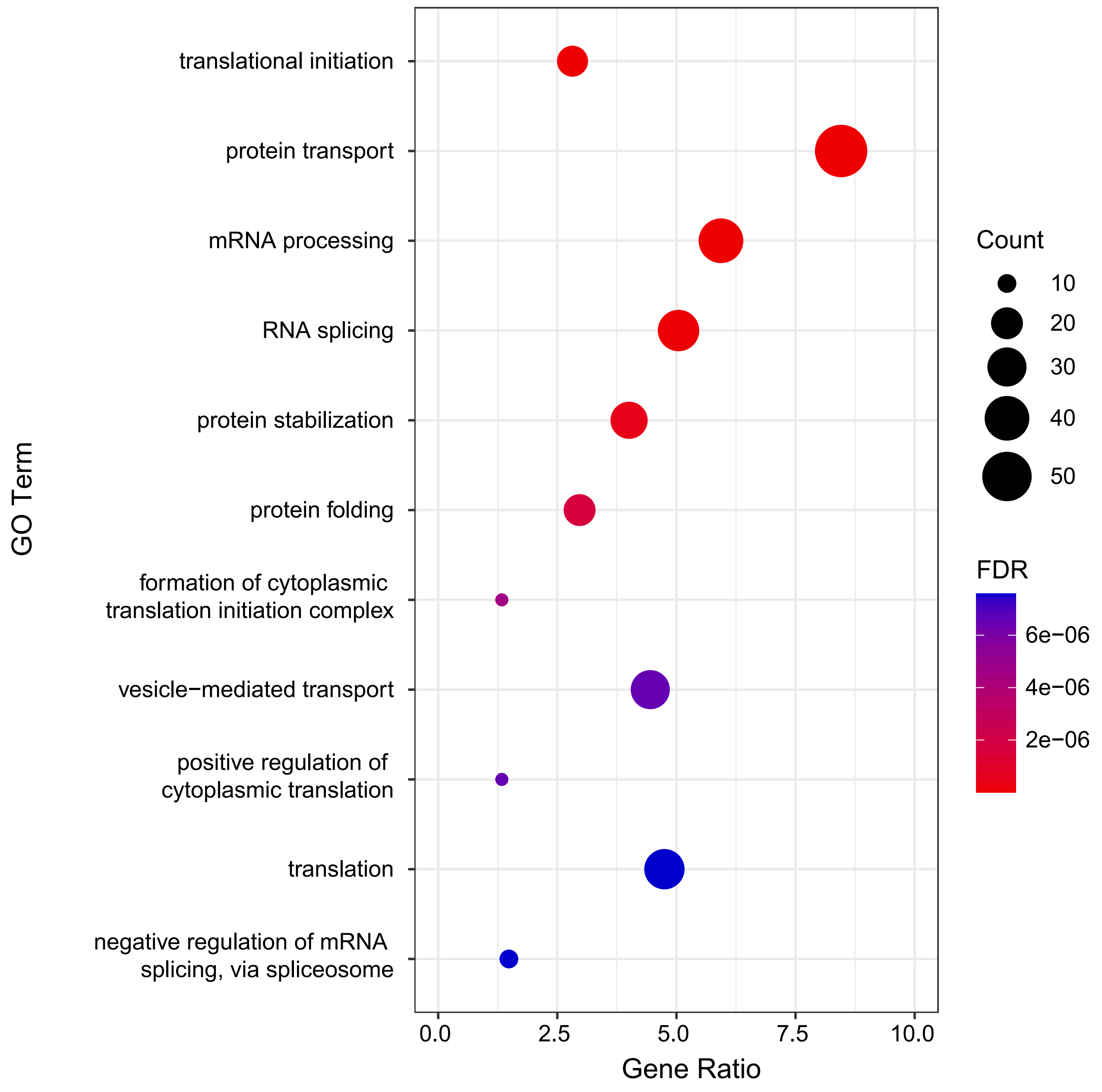

Fig. S3. Translation initiation-related genes were upregulated in protein level in CNOT7&8 KO oocytes. Results of GO enrichment analysis with upregulated protein in CNOT7&8 KO oocytes. Upregulated genes identified in proteome analysis were subjected to GO enrichment analysis with DAVID (https://david.ncifcrf.gov). Dot plot indicates the number of genes with each GO term and its ratio. Top 11 terms are shown.

Development • Supplementary information

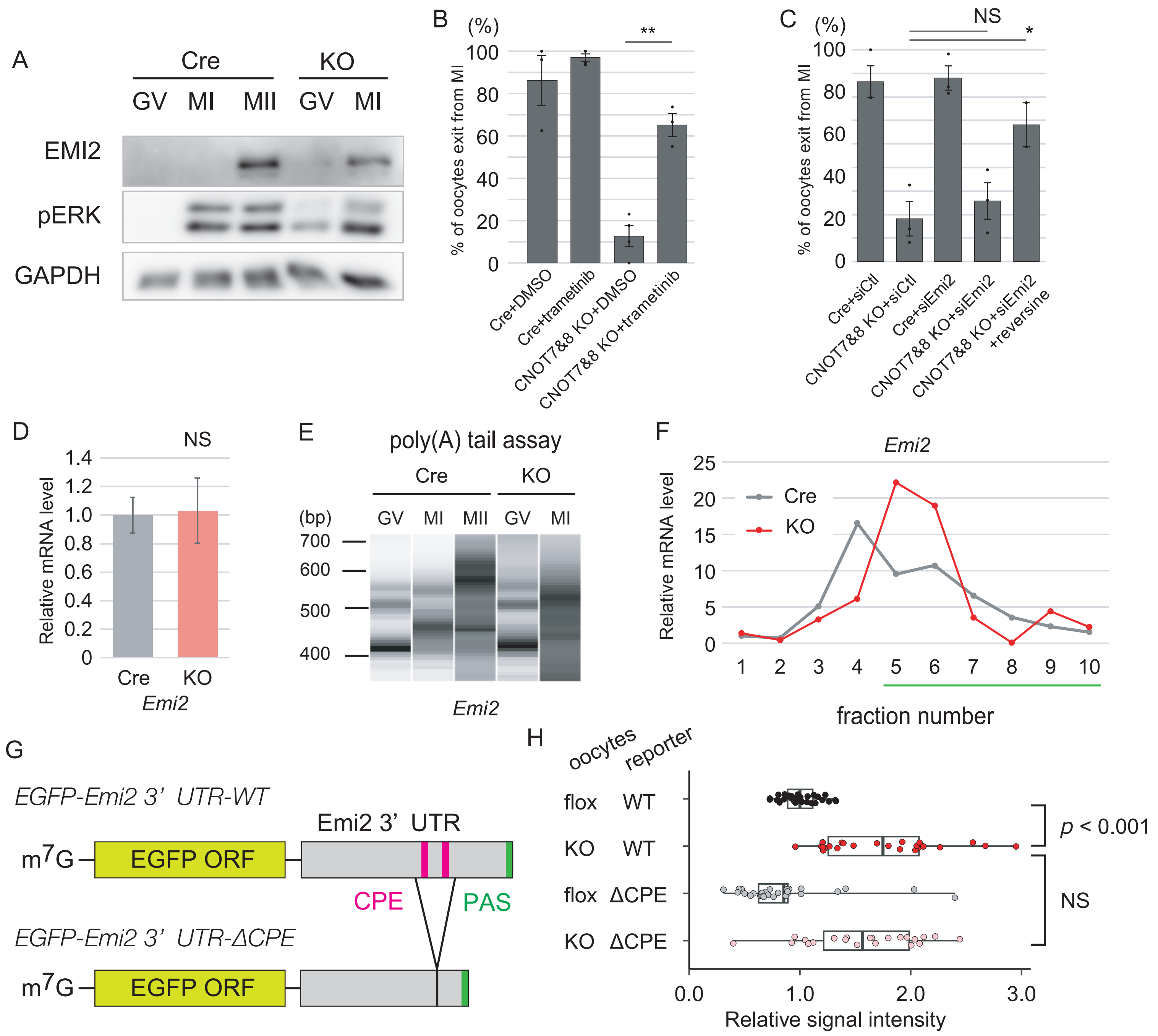

Fig. S4. Untimely translational activation of Emi2 in CNOT7&8 KO oocytes and its possible involvement in MI arrest. (A) Immunoblot images of Emi2 and phosphorylated ERK. Lysate of 30 oocytes was loaded in each lane. Representative images are shown. (B) Effect of trametinib on MI arrest in CNOT7&8 KO oocytes. The ratio of oocytes that exited from MI stage to oocytes that entered MI stage is shown. Average ratio of oocytes from more than three experiments is shown. n = 55 (Zp3-Cre+DMSO), 102 (Zp3-Cre+trametinib), 47 (CNOT7&8 KO+DMSO), 57 (CNOT7&8 KO+trametinib). **: p <0.01; Student' s t-test. Error bars indicate standard errors of the means. (C) Effect of Emi2 depletion on MI arrest in CNOT7&8 KO oocytes. The ratio of oocytes that exited from MI stage to oocytes that entered MI stage is shown. Average ratio of oocytes from more than two experiments is shown. n = 27 (Zp3-Cre+siControl), 32 (CNOT7&8 KO+siControl), 57 (Zp3-Cre+siEmi2), 62 (CNOT7&8 KO +siEmi2), 23 (CNOT7&8 KO+siEmi2+reversine). NS: not significant, *: p <0.05; Student' s t-test. Error bars indicate standard errors of the means. (D) Expression levels of Emi2 mRNA in

Development • Supplementary information

CNOT7&8 KO oocytes. mRNA levels were quantified by RT-qPCR. Bars indicate mean expression values relative to spike-in RNA. Error bars indicate standard errors of means. n = 3. NS: not significant: Student' s t-test. (E) Poly(A) tail assay on Emi2 mRNA. Poly(A) tail length was analyzed by poly(A) tail assay on Emi2 mRNA from the indicated stage. (F) Distribution of Emi2 mRNAs on polysome profile. Polysome fractionation by sucrose density gradient centrifugation followed by RT-qPCR was performed. Values relative to Cre Fraction 1 are shown. Green lines indicate polysome fractions. (G) Design of CPE reporter mRNAs. Magenta and green regions indicate CPE and polyadenylation signal (PAS), respectively. (H) CPE reporter assay in CNOT7&8 KO GV oocytes. CNOT7&8 KO or WT GV oocytes were injected with EGFP-Emi3-3' UTR-WT or ΔCPE, and RFP-poly(A). Each dot indicates relative EGFP signal intensity normalized to RFP signal. Horizontal lines indicate data range. Left, middle, and right vertical lines indicate lower quartile, median, and upper quartile respectively. p-value; Student' s t-test.

Table S1. Primer list

Available for download at
https://journals.biologists.com/dev/article-lookup/doi/10.1242/dev.201773#supplementary-data

Development • Supplementary information
